# *QuickStats*: Percentage* of Emergency Department Visits for Acute Viral Upper Respiratory Tract Infection^†^ That Had an Antimicrobial Ordered or Prescribed,^§^ by Metropolitan Statistical Area^¶^ — United States, 2008–2015**

**DOI:** 10.15585/mmwr.mm6703a7

**Published:** 2018-01-26

**Figure:**
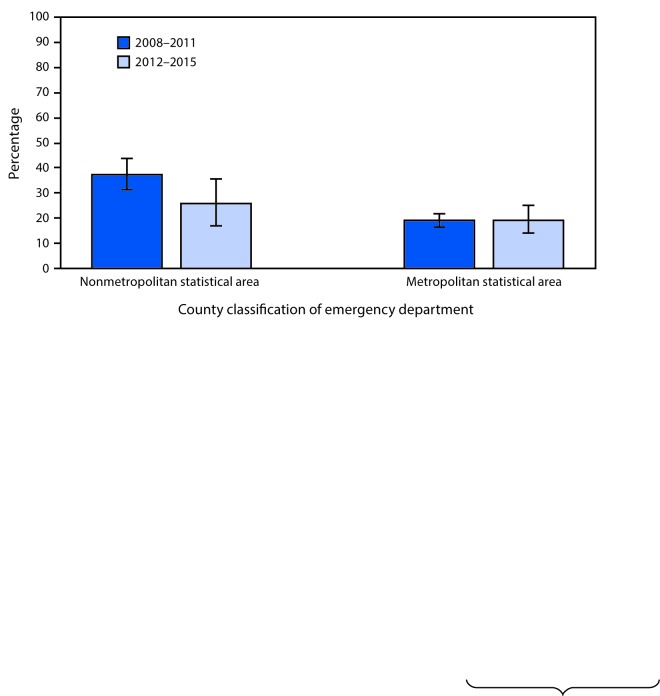
From 2008–2011 to 2012–2015, the percentage of visits for acute viral upper respiratory tract infection that had an antimicrobial ordered or prescribed decreased from 37.1% to 25.5% among emergency departments (EDs) located in nonmetropolitan statistical areas, but this decline was not seen among EDs in metropolitan statistical areas. In 2008–2011, the percentage was higher among nonmetropolitan EDs than metropolitan EDs, but there was no difference in 2012–2015.

**Source:** National Center for Health Statistics, National Hospital Ambulatory Medical Care Survey, 2008–2015. https://www.cdc.gov/nchs/ahcd/ahcd_questionnaires.htm.

